# Expectation and Reality: International Students' Motivations and Motivational Adjustments to Sustain Academic Journey in Chinese Universities

**DOI:** 10.3389/fpsyg.2022.833407

**Published:** 2022-02-24

**Authors:** Yuezu Mao, Hao Ji, Rujia Wang

**Affiliations:** ^1^Business School, Ningbo University, Ningbo, China; ^2^College of Electronic and Information Engineering, Ningbo Polytechnic, Ningbo, China

**Keywords:** motivation, push-pull factors, self-motivated pushing force, external pulling force, motivational adjustment

## Abstract

Considering the increasing influx of international students to Chinese universities in recent decades, it is surprising to find that few empirical research, especially longitudinal ones, have been conducted in exploring the motivation of international students in China. To fill up the existing gap, this study explored and tracked international students' motivations dynamically. Mixed research design, such as surveys, reflective journals, and interviews, was employed in this study. Data were collected from 671 international students and three teachers in three Chinese universities in Zhejiang province, mainland China on a longitudinal basis. The present study found that international students' motivation could be discussed with considerations to the following two different phases: preliminary phase before they come to China and follow-up phase when they are in China. This study found that the integrative understanding of the external pulling force and the self-motivated pushing force plays a vital role in answering international students' motivations to China. International students were driven more by the self-motivated pushing force than the external pulling force in selecting China as their study destinations. Moreover, international students experienced motivational changes when their expectations conflict with reality and their positive motivational adjustments and social interaction were important to the sustainability of their academic journey. Moreover, this study provides implications for the government, universities and international students in the aspects of policymaking, education and application.

## Introduction

China has become an important study destination for international students (Wu et al., 2019). Based on the report from the Ministry of Education of China, 492,185 international students from 196 countries were studying in Chinese universities in 2018, an increase of 80% compared with the figure in 2010 (Ministry of Education, 2010, 2018a). Given that China is not one of traditional international study destinations, it proposes a question: what factors motivate international students to come to seek education in Chinese universities, and how do they sustain their academic journey in China. Therefore, it is needed to investigate international students' motivations and motivational adjustments in the context of Chinese universities. The investigation into this aspect is not only beneficial for the greater good of international students, but also essential for the sustainability of international education in China.

The push-pull model has provided inspirations to understand international students' motivations while few empirical research, especially longitudinal ones, were conducted on international students' motivations in the Chinese context. Previous literature mostly concentrated on students' motivational trajectories to developed countries in the pursuit of educational programs taught in English (Wu et al., 2019; Gong et al., 2020) such as the US (e.g., Zhou, 2015) and the UK (e.g., Maringe and Carter, 2007). It is needed that international students' motivations to China should be integrated into the push-pull model as an important piece of jigsaw in adequately understanding international students' mobility. Moreover, previous studies have presented motivations to China as outcomes that remain relatively stable during international students' academic journey, while motivational changes and adjustment experienced by students were ignored (e.g., Jiani, 2017; Wen and Hu, 2019; Wu et al., 2019). It is problematic to assume motivation as a one-time static process in that it is unable to reveal a complete and dynamic picture of international students' motivations in China (Gong et al., 2020). Therefore, there is a pressing need that international students' motivations should be studied dynamically by looking into different phases of motivation.

To address the existing gap, this study aims to dynamically explore international students' motivations, and examine motivational changes they have experienced during their academic journey in China. Specially, this study aims to reveal the dynamic nature of international students' motivations through their interaction with the complex environment in the Chinese society. Moreover, this study also aims to enrich our understanding of the role of push-pull factors in motivating international students in the preliminary phase as well as motivational changes and adjustments in the follow-up phase.

## The Literature Review

### The Push-Pull Model and International Student Mobility

The push-pull model has been extensively applied to account for international student mobility (Li and Bray, 2007; Maringe and Carter, 2007). Before prospective international students move abroad, they are pushed away from their home countries by less favorable conditions while better opportunities in the host countries are pulling them (Altbach, 1998). A series of push factors contain limited educational opportunities (Mazzarol and Soutar, 2002), lack of employment chances (Altbach, 2004) and unstable domestic environment (Ahmad and Hussain, 2017). Accordingly, international students could be pulled or attracted by particular study destinations with better educational quality (Chen, 2007), good reputation (Shanka et al., 2006), employment prospects (Singh et al., 2014) as well as economic and social dynamics (Altbach and Knight, 2007).

While the push-pull model has provided a theoretical framework to understand international students' flow to Chinese universities, it still has limitations. Due to its overemphasis on macro-level factors and less attention to micro-level factors, the model has often come under criticism (Li and Bray, 2007). For instance, while China's economic development, employment opportunities, competitive quality of education, opportunities to learn Chinese culture and language are often mentioned as major pulling forces that attract prospective international students to seek education in China (Song and Liu, 2014; Jiani, 2017; Wen and Hu, 2019), a series of micro-level factors that drive international students internally are greatly neglected. The choice of studying abroad is largely a private decision though it could be influenced by external economic and sociocultural factors (Cantwell et al., 2009). Actually, a series of micro-level factors such as age, gender, motivations, academic abilities, financial status (Wen and Hu, 2019), personal attitudes, perceptions and desire could all influence individuals' decisions, choices and behaviors (Drugău-Constantin, 2019; Graessley et al., 2019; Meilhan, 2019; Mirică Dumitrescu, 2019; Pop et al., 2021; Rydell and Kucera, 2021; Watson and Popescu, 2021). The unbalanced ascription of international students' choices and decisions (in selecting educational destinations) to macro-level factors is misleading in reflecting the core structure of push-pull motivations (Wu et al., 2019). Therefore, it is important to pay more attention to the internal forces that motivate international students.

Furthermore, the push-pull model is problematic in explaining the mobility of international students to Chinese universities due to the homogeneous research samples collected. Most samples related to international student mobility are usually drawn from international students studying in developed countries (Heng, 2018), while motivations of international students pursuing education in other countries have rarely been investigated (Maringe and Carter, 2007; Singh et al., 2014; Jiani, 2017). Push-pull theories drawn from the unidirectional mobility, such as from developing countries (e.g., China, Brazil, India, Indonesia) to major developed countries (e.g., the US, UK, Canada, Australia), are incomplete in understanding the complexity in the global mobility of international students (Cantwell et al., 2009). Although some attention have been paid to non-western countries such as Mexico (e.g., Cantwell et al., 2009) and Malaysia (e.g., Singh et al., 2014), research on international student choice of China as destination still remain sparse and need to be integrated into the push-pull model. Consequently, a more integrated and balanced view is needed to focus on the effect and complexity of push-pull factors. Moreover, the heterogeneity of research samples could bring more complexity to international student mobility and the push-pull model.

Recent literature has advanced push-pull theories by paying more attention to the interplay of internal and external factors on students' choice of study destination (e.g., Jiani, 2017; Wen and Hu, 2019). For example, Wen and Hu (2019) has proposed a modified push-pull model that integrates macro- and micro-level analysis of educational, economic, sociocultural, and political factors to examine how individuals make study choice influenced by external factors. Another study conducted by Li and Bray has examined the interaction of individual factors (e.g., family background, academic characteristics, perceptions and motivations) and external factors (affordability, accessibility and desirability of higher education) in helping understand students' decisions in selecting Hong Kong or Macau as their educational destinations (Li and Bray, 2007). Although the above-mentioned studies suggest the use of the push-pull model in understanding international students' mobility, a dynamic view looking at the interplay of motivation and push-pull factors is still lacking (Gong et al., 2020). We consider it is significant to examine international students' motivations based on the interplay of internal pushing force and external pulling force, which can advance our understanding of the existing push-pull model and international student mobility.

### Motivation and International Students in China

The population of international students has been increasing rapidly in Chinese universities in recent decades (Wu et al., 2019). Till the present, China has become the largest receiving country in Asia and the third largest receiver worldwide (Ma and Wen, 2018). Despite the positive trend shown through numbers, research related to motivations of international students in Chinese universities is still scarce.

A few studies have probed into motivations and factors influencing international students' choices of China as study destination (e.g., Jiani, 2017; Wen and Hu, 2019; Wu et al., 2019) while research results remain contradictory. For instance, education quality of a destination country was considered as the main pull factor in international student choice of study destination (Wei, 2013). Nevertheless, it was not the case for international students in Shanghai as some higher education institutions in Shanghai have not earned world renowned reputations that are attractive to international students (Ding, 2016). Similarly, the relatively low tuition fee and living cost could attract international students to come to China for affordable education (Liu et al., 2013) while it may not be the case for students who are better-off financially as they could be more motivated by their desire for leaning Chinese culture and language (Jiani, 2017; Gong et al., 2020). In real situation, motivations could be intertwined and act like dynamic mixture, which will be later explained in the discussion part of this study.

Furthermore, it is obvious that the above research examines motivations from a relatively static perspective by investigating students' motivations to China. However, research, in this regard, is too simplistic to capture the variability and complexity of motivation with an emphasis on international students (Gong et al., 2020). Therefore, aside from the investigation of international students' motivations to China, more attention should be focused on motivational changes and adjustments experienced by international students when they are in China.

In general, gaps exist in prior literature with respect to international students' motivations of studying in China, which could affect our observation of the real picture of motivational phenomena. Considering that studying in China has become an increasingly attractive option among prospective international students (Wen and Hu, 2019), it is of necessity to observe motivations of international students in a developmental perspective. To achieve academic success in an intercultural environment, acculturating individuals need to make changes or adjustments at different levels, and the question confronting them is not whether they adjust or not, but how and to what extent they adjust (Berry, 2005). However, research associated with student sojourners' motivational changes and adjustments remains marginal (Gong et al., 2020). More inquiry is needed to examine changes in international students' motivations and their reactions to such changes.

## Methods

### Participants

Participants of the survey were selected from three Chinese universities in Zhejiang province. The questionnaire was delivered through the researchers' direct contacts or colleagues from student affairs offices in other universities. Before the study, all participants were guaranteed that the data collected would be held anonymous and confidential. In total, 688 participants completed the questionnaire, among which 671 pieces of valid surveys were collected. The demographic information of participants is presented in Table 1. The descriptive analysis results by SPSS 23.0 have demonstrated that the number of males (367) is slightly more than that of females (304). The age range of participants is between 17 and 33, and they are mostly from undergraduate and master programs. The demographic figure of participants is in line with the general attributes of the sample universities, which is thus appropriate for this study.

**Table 1:** Demographic information of the survey (*N* = 671).

**Items**		***N* (%)**	**Items**		***N* (%)**
Gender	Male	367 (54.7)	Time in China		
	Female	304 (45.3)		1–6 months	187 (27.9)
Age	17–20	250 (37.3)		7–12 months	36 (5.4)
	21–24	326 (48.6)		1–2 years	146 (21.8)
	25–33	91 (13.6)		2–3 years	136 (20.3)
Study Program	Undergraduate	597 (89.0)		3–4 years	98 (14.6)
	Master	59 (8.8)		Over 4 years	68 (10.1)
	Doctoral	2 (0.3)	Origins		
	Exchange	13 (1.9)		Africa	338 (50.4)
Discipline	Business	329 (49.0)		Asia	261 (38.9)
	Medicine	104 (15.5)		Europe	47 (7.0)
	Science and Engineering	78 (11.6)		America	13 (1.9)
	Humanity, Law, Language, Art	30 (4.5)		Oceania	12 (1.8)
	Others	130 (19.4)			

A total of 19 participants took part in reflective journal writing and interview on a longitudinal basis. All the interviews were recorded and transcribed with permission from participants. Meanwhile, to protect participants' interests and ensure they are not exposed to violation of privacy or loss of dignity, the informed consent has been obtained throughout the whole research (Cohen et al., 2017). Each interview lasted between 20 and 80 min. Altogether, we received 106 pieces of reflective journals and conducted 35 in-depth interviews between March, 2020 and July, 2021. Their profiles could be seen in Table 2.

**Table 2:** Participants' profiles.

**No**.	**Name**	**Age**	**Gender**	**Home country**	**Major**	**Category**
1	Lily	21	Female	Rwanda	Business administration	Student
2	Sally	20	Female	America	Medicine	Student
3	Sandra	30	Female	South Africa	Business administration	Student
4	Jessica	25	Female	Antigua and Barbuda	Chinese language	Student
5	Rachel	25	Female	Zimbabwe	International trade	Student
6	Cindy	23	Female	Indonesia	International trade	Student
7	Anna	22	Female	Indonesia	Business administration	Student
8	Mary	21	Female	Morocco	Logistics management	Student
9	Kelvin	24	Male	Ethiopia	Architecture	Student
10	Yassin	26	Male	Central Africa republic	Business Administration	Student
11	Gutlev	24	Male	Russia	Law	Student
12	Yusuf	25	Male	Tanzania	Business administration	Student
13	Connie	24	Male	Germany	Business administration	Student
14	Gokul	27	Male	India	Medicine	Student
15	Sammy	23	Male	Russia	Business administration	Student
16	Welly	24	Male	Yemen	Engineering	Student
17	Jenny	28	Female	China	English	Recruitment Officer
18	Yuki	29	Female	China	English	Student Councilor
19	Hulk	35	Male	China	Finance	Lecturer

*Participants were all given pseudo names*.

### Instruments and Procedure

The current study adopts mixed methods to collect data from three different sources, namely a self-reported motivation questionnaire, reflective journals and interviews. The questionnaire measured the degree of international students' motivations and push-pull factors received. The reflective journal and interview aimed mainly to understand motivational changes and adjustments experienced by international students. This study uses multiple data source to triangulate, which is to increase reliability of data and compensate between strengths and weakness of the research (Denscombe, 2014).

To identify the attributes of motivations and the strength of push-pull factors, this study has employed the quantitative approach in the first half of study. The motivation scale was revised from Yang (2005) and Zhu's (2011) questionnaires on motivations of international students to China, including three parts, respectively: survey introduction, demographic information part, and the measurement of 12 motivation items. An example motivation item is, “I want to learn and improve my Chinese.” The Likert scale was used (1 = Not important, 5 = Extremely important) with a higher score indicating a stronger motivation. The 12 items include students' motivations in aspects such as academic pursuit, language acquisition, ability improvement, and personal development. The Cronbach's alpha value of this scale is 0.825, which is highly reliable.

To track motivational changes and adjustments international students' experience during their academic journey in China, this study has adopted the quantitative approach in the second half of study. International students were asked to keep reflective journals on a voluntary basis. When writing reflective journals, international students could experience a recall of the experience, a revisit of context and situation, allowing them to reflect, think and learn critically (Raterink, 2016; Draissi et al., 2021). Besides, some questions regarding their motivations were recommended to guide participants' reflective journal writing, such as: *What has motivated you to come to China to study? What factors have influenced your choice of China as study destination? And did you find any differences between your expectation and reality?*

Aside from reflective journals, interviews were also conducted to track the trajectory of students' motivational changes. Interviews for students mainly included questions about students' demographic information, academic performance, language level, motivation to China, as well as general feeling about their intercultural experience. As a longitudinal study, there were often follow-up interviews with our participants, and the follow-up interviews were mainly concerned with questions about difficulties and problems they encountered, their changes of motivations, and their adjustments during their academic journey in China. In addition, interviews for teacher and staff member were about cases they met during their teaching and management experiences.

### Data Analysis

Descriptive analysis, using SPSS26.0, was conducted to measure the strength of international students' motivations represented through means and standard deviations of the variables. Then, principal components analysis was conducted to obtain smaller number of constructs and examine the key dimensions of motivations of international students to China. Spearman correlations were calculated to examine bivariate associations between motivational variables. To tell the differences and strength of push-pull force, a paired sample *t*-test was conducted. A paired sample *t*-test is referred as *t*-test for correlated samples, which suggests it is a test to tell differences between two correlated groups.

All reflective journals and interview transcriptions were read recursively, and thus coherent representation of the data and their meanings can be presented (Cohen et al., 2017). NVivo 12 was adopted for exploring qualitative data. An open-coding approach was applied, and open codes such as “types of motivation,” “influential factors,” “motivational changes,” “motivational adjustment,” and “plan after graduation” were identified, under the framework of the push-pull model, aiming to reduce massive data to relative categories. For example, texts like “good quality of education” and “my father recommended me to come to China” were categorized as “pull factors”, while “teaching in my home country is not as good as here” and “conflicts in my country” were classified in the “push factors” category.

After identifying various stages and categories of motivations, the selective coding was applied to identify the core category around which other sub-categories were related and integrated (Strauss and Corbin, 1982). For example, the sub-categories “pull factors” and “push factors” were integrated into “preliminary phase: motivation to China”, while “motivation gaps” and “motivational adjustments” were subsumed under the category of “follow-up phase: motivation in China”. Coding in such way facilitates the authors to find participants' motivational adjustment process and influential factors. Meanwhile, the creation of such hierarchy is mainly to keep our findings tidy and clear, and in the meantime ascribe meaning to the data (Gibbs, 2007). This study has borrowed some codes existing in the literature, but it also modified and deleted some codes when new categories emerged, thus better explaining research findings.

## Results

Data analysis has demonstrated that international students experienced various motivational changes in their transition from home countries to host countries. To examine such changes, motivations should be examined dynamically based on the following two different phases: preliminary phase before they come to China and follow-up phase when they are in China. Survey results have shown that international students were driven more by the self-motivated pushing force than the external pulling force in their choices of studying in China. Moreover, the qualitative analysis results indicate that motivational changes and adjustments during the second phase are vital to the sustainability of international students' overall academic journey in China.

### Preliminary Phase: Motivation to China

#### What Motivated International Students to China

Descriptive analysis was first applied to examine the strength of international students' motivations. The analysis has shown that the mean values of all items were above 3.5 (see Table 3), indicating that international students considered all motivations to China as important or very important. Eight items were marked by the participants as very important or above, while the rest four items were marked as between important and very important. Besides, no items were considered by participants as not important. The three most important motivations of international students are “Complete study and get diploma,” “Study overseas for better personal development,” and “learn and improve Chinese”, while the three lowest ranking motivations are “Easier admission,” “Low tuition fee,” and “Better education in Chinese universities.”

**Table 3:** International students' motivations to China (*N* = 671).

**No**.	**Item**	**M**	**SD**
1	Complete study and get diploma	4.687	0.713
2	Study overseas for better personal development	4.656	0.653
3	learn and improve Chinese	4.575	0.765
4	Improve knowledge in professional fields	4.563	0.728
5	Broaden my horizon and enrich life experience	4.553	0.731
6	Tour around and know Chinese culture	4.504	0.784
7	Train myself in adapting to different cultures	4.498	0.776
8	Got scholarship	4.110	1.247
9	Recommendations from parents/friends	3.955	1.129
10	Better education in Chinese universities	3.756	1.090
11	Lower tuition fee	3.663	1.108
12	Easier admission	3.545	1.040

In general, the twelve items can be further categorized into push-pull factors in accordance with the push-pull model. A series of push factors which drives international students internally include “Complete study and get diploma,” “Study overseas for better personal development,” “Learn and improve Chinese,” “Broaden horizon and enrich life experience,” “Improve knowledge in professional fields,” “Tour around and know Chinese culture,” and “Train myself in adapting to different cultures.” By contrast, the pull factors involve “Scholarship opportunities,” “Recommendations from parents/friends,” “Better education in Chinese universities,” “Lower tuition fee,” and “Easier admission.”

From the above analysis, it can be found that international students come to China with various motivations that mainly include educational, economic, sociocultural. Educational motivations contain “Complete study and get diploma,” “Study overseas for better personal development,” “Learn and improve Chinese,” “Improve knowledge in professional fields,” “Better education in Chinese universities,” and “Easier admission,” which have more pushing factors than pulling factors. Similarly, sociocultural motivations have more items in pushing than pulling students, which include “Broaden horizon and enrich life experience,” “Tour around and know Chinese culture,” “Train myself in adapting to different cultures,” and “Recommendations from parents/friends.” On the contrary, economic motivations act as major pulling forces including “Scholarship opportunities” and “Lower tuition fee.”

### The Influence of Push-Pull Factors on Motivations

To examine the key constructs of motivations of international students to China, principal components analysis was conducted. Eigenvalues equal to or >1.00 were extracted, and the indicators (KMO = 0.892, Bartlett's test *p* < 0.001) showed that the data were appropriate for performing factor analysis. From the twelve variables that were analyzed, orthogonal rotation of the variables yielded two principal factors, accounting for 36.429 and 17.978 percent, respectively, and accumulatively explaining 54.407 percent of the total variance. The factor loading and Cronbach's alpha values, are presented in Table 4. The items with a factor loading larger than 0.42 were selected. Based on the contents of the variables, the two main factors are named as “Self-motivated pushing force” (F1) and “External pulling force” (F2), respectively.

**Table 4:** Principal components analysis (*N* = 671).

**Factors**	**Items**	**F1**	**F2**
F1: Self-motivated pushing force	Learn and improve Chinese	**0.558**	0.252
	Complete study and get diploma	**0.709**	0.164
	Study overseas for better personal development	**0.813**	0.160
	Tour around and know Chinese culture	**0.821**	0.180
	Train myself in adapting to different cultures	**0.805**	0.144
	Improve knowledge in professional fields	**0.817**	0.115
	Broaden horizon and enrich life experience	**0.841**	0.105
F2: External pulling force	Got scholarships	0.192	**0.623**
	Lower tuition fee	0.045	**0.736**
	Easier admission	−0.018	**0.791**
	Better education in Chinese universities	0.287	**0.422**
	Recommendations from parents/friends	0.280	**0.480**
	**Eigenvalue**	4.371	2.157
	**Cronbach's alpha**	0.891	0.641
	**% of variance**	36.43%	17.98%

*The bold values are factor loading*.

The two extracted factors can be understood as the two categories in the push-pull motivations model. The push factors involve motivations to broaden horizon and enrich life experience, know Chinese culture, improve professional knowledge, gain personal development, acquire intercultural training, obtain academic success, and learn Chinese, which drives international students internally to seek education in China. The pull factors are more associated with external factors that could attract international students, which consist of scholarship opportunities, lower tuition fee, easier admission, better education quality, and recommendations from others. Briefly, the two extracted principal factors could represent the push-pull factors, influencing international students' motivations to China both internally and externally.

To know the relative power of push and pull factors on motivations, the 12 items were transferred into the two identified factors (Self-motivated pushing force and external pulling force) by their mean values. The results of the *t*-test analysis demonstrate that self-motivated pushing force and external pulling force are statistically significantly different (*p* < 0.001) motivational factors. Moreover, the correlational significance level of 0.419 (*p* < 0.001) indicates that the two motivational factors are significantly related, and the effective size (Cohen's d = −1.8051) represents a large effect of difference (Cohen et al., 2017, p. 746). Self-motivated pushing force (mean = 4.577) is stronger than the power of external pulling force (mean = 3.806), which represents that the international students were driven more by the self-motivated pushing force when compared with the external pulling force.

### Follow-Up Phase: Motivation in China

#### Motivational Changes: Expectation and Reality

By comparing participants' motivations before and after they came to China, it can be concluded that most of the participants, mentioned that they experienced motivational changes through reflective journals and interviews (see Table 5). Totally 12 participants have reported that they changed their preliminary motivations and made relevant changes after they spend some time in China, except for 3 students who did not directly mention their motivational changes. By performing the data analysis, we found that participants' motivational changes mainly derived from their inappropriate expectation or misjudgment of reality.

**Table 5:** Cases of motivational adjustments among international students in China.

**No**.	**Name**	**Preliminary motivation**	**Follow-up motivation**	**Motivational adjustments**
1	Sandra	Find a job in China	Continue her study before getting employed	From economic to educational
2	Jessica	Look for educational opportunities	Learn Mandarin and experience Chinese culture	From educational to sociocultural and educational
3	Mary	Experience new culture and start business someday	Adjust to academic pressure and tasks	From cultural and economic to educational
4	Anna	Find part-time jobs	Focus on study	From economic to educational
5	Kelvin	Come to seek education	Look for fun and pleasure	From educational to sociocultural
6	Connie	Study accounting, learn mandarin	Study accounting and postpone mandarin learning after graduation	Decrease of educational motivation
7	Sammy	Study mandarin, start business, experience Chinese culture	Focus on study and learn mandarin	From mixed to educational
8	Welly	Experience different education, search for real knowledge	Support himself financially	Educational to economic
9	Lily	Her sister suggested to come to China	Get enough credits and graduate	Sociocultural to educational
10	Sally	Family members are in Ningbo	Get diploma, make more friends, start business	Sociocultural to mixed
11	Yusuf	Know more about Chinese education and economy	Learn language for one more year	Educational and economic to educational
12	Gokul	Receive medical educational and become a doctor	Pass doctor license exam and find job in China	Educational to mixed

*Participants were all given pseudo names*.

International students usually come to China with expectations while they often find their expectations inappropriate when they confront unexpected reality in China. Meanwhile, their preliminary motivation could not be sustained. When asked about the reasons to come to China, Connie (male, Germany) mentioned that he came to China mainly to “study accounting and learn the Chinese language”. However, his motivations turned out to be too ambitious after he came to China, finding that “I underestimated how difficult accounting would be, so I had to devote more time to study accounting and had less time studying the Chinese language.” Obviously, the gap between Connie's expectation and reality has influenced him to adjust motivations and concentrate on learning accounting rather than the Chinese language in order to reach sustainable academic goal. Nevertheless, Connie did not forget his preliminary motivation. During the last interview 1 year later, he mentioned that:

After graduation, I still want to do one language semester to polish my Chinese, and then I will try to do an internship probably in an area where the Chinese language is still needed. (Connie, male, Germany)

Although international students make expectations about practical situations in China, misjudgment happens when their prior knowledge is not sufficient. Sandra (female, South Africa) has expressed her preliminary motivation to China is “for employment because her husband got a job in China” without the investigation into the Chinese labor market beforehand. After coming to China with her husband, she noticed the gap between her qualification and the demand of job market, which explains why she needed to give up her preliminary motivation for employment and made corresponding adjustments as she mentioned through interview that:

Once I got here, I found out that I needed a degree to be able to work, so that's why I chose this university. It was close to where we live. (Sandra, female, South Africa)

Without doing enough homework on China's job market, Sandra encountered difficulties and described her preliminary motivation of getting employed is:

Very big accomplishment to achieve, and it's not as easy as I think in most other countries and it's a very competitive market. (Sandra, female, South Africa)

Nevertheless, the adjustment worked out well for Sandra. One year later when she accepted another interview, she mentioned to the first author about her new motivations:

My new motivations are to complete the study here and to get employed in the future, and then to start my own company. I have made great progress compared with the first year I came. In fact, I may graduate earlier. I think the key here is stayed connected with the school and people, and always remain positive attitudes in any situation. (Sandra, female, South Africa)

It is obvious that Sadra has experienced motivational changes during her academic journey. She has shifted from economic motivation to educational motivation, and back to economic motivation in the future. For her, all these motivational changes are approaches to strike a balance with the upcoming educational pressure.

Additionally, it also needs to be mentioned that her integrative attitude toward the new environment is important to keep herself motivated educationally. Such result is in accordance with Ward and Berry's finding that the acculturative attitude of integration is considered to be the most positive and favored result for international students in order to fit in a new environment (Ward, 2001; Berry, 2005). In fact, the motivational change has provided Sandra with the new driving force to overcome difficulties positively and continue study.

Overall, it is common that international students make necessary motivational changes when they find gaps between expectation and reality. From Connie and Sandra's academic experiences, it could be observed that international students experienced active motivational changes so as to sustain their academic journey. Actually, international students could have multiple motivational changes to narrow the gap between expectation and reality during their whole academic journey. In this sense, international students' awareness of sensing conflicts between expectation and reality could provide a good chance for them to reflect on and develop themselves. In real life, it could be hard for them to make exact expectations when travel between intercultural destinations. When students find challenges and difficulties that are not expected, it is suggested that students make timely motivational changes to find alternative solutions in order to cope with new challenges and realize sustainable development.

### Motivational Adjustments Through Intercultural Experiences

International students' intercultural journey is not without risks and challenges, and their timely positive motivational adjustments are vital to the sustainability of their academic journey. Data analysis from interviews and reflective journals has concluded that international students' motivations can be influenced by their changing attitude, knowledge, ability and skills, as well as identity throughout their academic journey in China.

Taking Jessica's case an example, it can be found that she experienced motivational adjustments from educational to sociocultural and educational. Like many other students, Jessica mentioned that her preliminary motivation to China was educational. She wrote in the journal that:

I guess what motivated me to come to China study was the educational opportunity that I was looking for when it had presented itself, being that I was selected as a recipient of the CSC Scholarship. (Jessica, female, Antigua and Barbuda)

However, Jessica' preliminary motivation to China as a scholarship winner did not necessarily lead to a smooth intercultural experience. Meanwhile, her experience in China was not satisfying as she recalled during the interview:

The first few weeks when I arrived in China, I was feeling isolated. Most lecturers' teaching style includes the use of PPT, and they tend to go at a fast pace. What worried me the most was, not being able to understand the content being taught in the classroom and understand grade-based assignment. These problems, I did not want to tell my parents, and most time, I ate alone and walked alone, I was not able to perform good on study and it was a bad memory. (Jessica, female, Antigua and Barbuda)

Based on Jessica's feelings, emotions and behaviors, we can observe that she encountered huge difficulties during her first several weeks in China. Unfamiliar environment, different teaching style, new academic requirement and being away from home were all push factors that have influenced Jessica's educational motivation. As a result, she kept herself away from contact with family members and friends. However, it was not the end of story for Jessica as international students' emotion, attitude, and behaviors changed over time (Gorard, 2001). Half a year later, she shared a different feeling in her journal:

After being here long enough, I came to realize that even though everything here is different, the culture in its entirety is not so strange. There are lots of interesting activities and beautiful places, you need to be adventurous or outgoing to enjoy. Studying in China has taught me to become an explorer and be able to discover the curiosity and excitement that China holds. Now I have new aims, like, to learn Mandarin, appreciate other cultures and overcome challenges of living in another country, and gain a greater understanding of the Chinese culture. I think I have got used to my study and life in China now. I have both Chinese friends and friends from other countries. I share feeling with my parents, they are also interested in listening to my stories here. (Jessica, female, Antigua and Barbuda)

Through Jessica' words, we can find her feelings, knowledge and attitudes toward China and the Chinese people have been changing through interesting cultural activities she participated and beautiful places she visited in China. The interaction with the host society has stimulated her positively and enabled her to regain educational motivation and develop sociocultural motivation.

Although the case of Jessica has set a successful example concerning motivational adjustment, motivational adjustments do not lead to positive motivational adjustments. Speaking of preliminary motivation to China, Welly mentioned during the interview that:

I wanted to go abroad because I see the education system in my country is not what I'm seeking for, so I seek to go abroad and search for real knowledge so that I can benefit from that. (Welly, male, Yemen)

He was admitted into the first university in China while his first academic experience in China did not turn out to be successful when he found that “I could not progress in the first university because of my poor English level.” Additionally, he also mentioned during interview that:

I totally changed my perspective, so since then I never got money from my father. I never was financed by my father, I was only financing myself, and I find myself a hobby that I can earn money from. That is about sneakers. So especially like Nike, Adidas, these things people have hype for them. So, if you buy them in retail, they will create a value in a resale market. So that's how I make (money), how I lived. (Welly, male, Yemen)

Obviously, the sudden cut-off of financial support from his father, though the reason was not mentioned, generates a significant influence over Welly's educational motivation. To support himself in China, he lost his preliminary educational motivation and adjusted to a more profitable economic motivation. Certainly, such motivational adjustment is not a good result for Welly to complete his study. At the same time, it has remained unclear whether Welly could make motivational adjustments again.

The findings presented above have demonstrated that positive motivational adjustment is of critical importance to the sustainable development of students' academic journey. Without positive motivation, students' academic journey could not be sustained. The viewpoint was also agreed by Yuki, an international student councilor, who shared a case with the first author during the interview:

I knew a student who was deported back to his own country because of drug problem. He was found smoking marijuana in his dorm. He skipped lots of classes and often hung around with some friends in night bars. I think China is only a free place for him and he lost motivations to study here. (Yuki, female, China)

According to the case provided by Yuki, the student failed his study in China without making accordingly positive motivational adjustment. Educational motivation is of great importance to sustain students' academic journey. When educational motivation is lost, academic failure for international students could not be avoided. As a result, social support received by international students may be of great importance. Hulk, a lecturer of the course “Strategic Management”, mentioned a student through interview:

The student was not focusing on study at first, he rarely attended my classes, so I asked the monitor to send a warning to him. He came to me and told me that he spent a lot of time doing part-time work here, and I had a long talk with him and urged him to put study before making money. After that conversation, I saw him more often on classes. (Hulk, male, China)

Based on Hulk's words, we can find the teacher's influence generated a positive impact over the student's motivation, which has helped the student adjust from non-academic motivations to educational motivation. Teachers' support, in this sense, fostered the students' educational motivation.

Therefore, to achieve greater academic success and to graduate, it is essential that international students establish positive social interactions in the host society. Therefore, they can draw on resources to stay positively motivated on educational tasks and sustain their academic journey in China.

## Discussion

### Motivations and Integrative Push-Pull Factors

The present study believes that the mobility of international students to Chinese universities should be integrated into the push-pull model. Prior literature has adopted a perspective on push-pull factors usually considering push factors as unfavorable conditions from the home country (Yu, 2010; Ma and Wen, 2018) and pull factors as favorable conditions from the study destination (Mazzarol and Soutar, 2002; Maringe and Carter, 2007). The perspective has often been used to explain the mobility of international students to developed countries (Wu et al., 2019; Gong et al., 2020) while it is inadequate in explaining the flow of international students to less-developed countries.

Educational market in China has its own traits to add to the push-pull model, and more attention should be paid to this emerging educational hub in Asia. For example, Bodycott (2009) has found that educational qualifications (e.g., diploma) gained from a western developed country is considered as a prestige for international students, accounting for why international students are attracted to developed countries to seek education. However, it may not be the case for international students in China as some students came with economic or sociocultural motivations rather than educational reasons (Ding, 2016). The inconsistent results show that a more comprehensive view needs to be taken. Based on the research findings, international students are diversified in their motivations, and educational motivation is not the sole pulling force (Wen and Hu, 2019). Economic and sociocultural motivations could also attract international students. Moreover, international students could be more driven internally rather than externally, which explains why the importance of external pull factors should not be overly emphasized. Therefore, this current study has proposed that a mixture of motivations and integrative push-pull factors have brought international students to Chinese universities.

The use of the push-pull model in explaining mobility of international students is not new while the model is often critiqued due to its overemphasis on macro-level pull factors (Li and Bray, 2007). This study found that, pull factors, though played a stronger role in motivating students, work with internal pushing forces to exert integrative influence over students' choices of study destinations. The quantitative part of this study investigated the attributes of motivations, concluding that external factors such as attractiveness of alien culture, easier admission, lower tuition fee, scholarship opportunities, better education quality, and parents' suggestions could play as main pull factors attracting international students, which is in line with prior literature (e.g., Mazzarol and Soutar, 2002; Yang, 2018; Wu et al., 2019).

However, after comparing the influence of push-pull motivations, this study has found students' pursuit of educational chances and future development (e.g., complete study and get diploma, improve Chinese, learn new knowledge) served as internal drivers and the major pushing force that motivated them to come to China. Self-motivated pushing force is stronger than the power of external pulling force, which represents that the international students were driven more by the self-motivated pushing force when compared with the external pulling force. The stronger strength of pushing factors over pulling factors in attracting international students has been largely neglected in the previous literature. In fact, more emphasis should be given on the micro-level aspects of the push-pull model (Wen and Hu, 2019). The results obtained from this study indicated that a balanced stance needs to be taken to evaluate the effect of push-pull factors. At the same time, both macro-level pull factors and micro-level push factors should be integrated, and thus a complete and comprehensive picture concerning motivations of international students could be unveiled.

Findings of this study can provide implications to educational practice in China in the following aspects. At first, the present study suggested that China's favorable policies and educational conditions are of critical importance in pulling and motivating international students, which is consistent with prior studies (Wen and Hu, 2019; Wu et al., 2019). As a result, the Chinese government and universities should improve existing policies and educational conditions to provide a better environment for the sustainable development of international education in China. Second, although previous studies have suggested that international students could be attracted by external pulling forces such as China's booming economy, employment opportunities, and sociocultural influence (Liu et al., 2013; Yang, 2018), their choice of China as study destination is the result of overall considerations with self-motivated driving force playing a stronger role. Consequently, China and its universities should continue to enhance its soft power (Yang, 2010; Tian and Lowe, 2018), and improve international students' academic experiences in order to attract more high-level international students that is consistent with China's future educational plan (Ministry of Education, 2018b). Finally, prospective international students should be more prepared in attitude, knowledge, skills, and abilities. Besides, international students should set appropriate motivations, as well as be ready for motivational changes and adjustments in their academic journey in China.

### Motivational Adjustments Through Intercultural Interaction

The research findings show that international students' motivations go through changes and adjustments, shaped through their interaction with the new environment. Their motivational changes and adjustments are closely associated with the changing gap between their expectation and reality, as well as with their increasing intercultural experiences. Thus, a dynamic and interactive view is necessary to be taken to comprehend the ever-changing characteristics and the evolvement of international students' motivations. This result is line with findings with prior studies (Wu et al., 2019; Gong et al., 2020).

Traveling from their own countries to China, international students do not always keep their motivations static. Entering a new cultural environment, international students make dynamic adjustments motivationally to overcome intercultural difficulties and problems. The existing difficulties and problems, if not dealt properly, will influence intercultural journey of international students (Lee and Rice, 2007; Chiang, 2015). Thus, students' motivations should not be observed from a static perspective. Their motivations transited with the changing environment to achieve academic goals and sustain personal development while the phenomena have rarely been discussed in the literature (Gong et al., 2020). Study in this aspect is, in fact, of great importance to understand the dynamic characteristics of students' motivations. This study has found that when international students encounter problems in real life when they have inappropriate expectations and misjudged situation in China before they came, their motivational adjustments are necessary steps to sustain their academic journey. Students' preliminary motivations often provide them with initial driving power influenced by integrative push-pull factors, whether they can complete their study plan is largely dependent upon the success of their adjustment in their follow-up motivations when they interact with the Chinese society and people. The result is consistent with Gong and his colleagues' study on New Zealand students' motivational shifts during their leaning of Chinese in China (Gong et al., 2020), while with a broader application by investigating international students' whole academic journey.

An interactive view is taken in this study in that international students' motivations could be influenced by their interaction with the host society. International students' intercultural journey in China is full of surprises and problems. This process could involve affective fluctuation, behavioral shifts, and cognitive changes (Ward, 2001). Language barriers, academic pressure, and communication problems can all bring difficulties and dilemma to them (An and Chiang, 2015; Yang, 2018). As a result, their motivations could experience changes (e.g., enhancement, decline, loss, regaining) in order to survive the new challenges. The case of Jessica has demonstrated that intercultural interaction could stimulate international students positively and help them enhance or regain educational motivation. The finding is consistent with studies by Gong et al. (2020) and Du and Jackson (2018), indicating that international students experienced motivational shifts after relocating to the new educational settings in order to sustain their academic journey.

A positive relationship between international students and the host society is conducive to the sustainability of students' educational motivations. According to Berry, different attitudes acculturating individuals take will greatly affect the results of individuals' adjustment to the local society (Ward, 2001; Berry, 2008). In this sense, when international students adopt the integration attitude toward the Chinese people and society, it is probably that they engage more in interaction with teachers and students who can provide more social support. Therefore, positive interaction between international students and the host society is more likely to yield strong international driving forces that motivate students to obtain academic success and achieve sustainable personal development. Moreover, Emadpoor et al. (2015) pointed out that educational motivation had a directly and positively relationship with the perceived social support. This conforms to the finding of this study that participants exhibited stronger motivation in their academic tasks when they receive more support from teachers and universities.

Additionally, this study has also found that the result to motivational adjustment is not always successful, and that support from university and teachers is of critical importance to the sustainability of international students' academic journey. Therefore, Chinese universities and teachers should pay more attention to educational reputation, teaching facilities and quality, disciplinary setting and criteria, living conditions as well as service level, and thus international students can be motivated more positively to complete their study in China. The way international students are taught and cultivated should be more student-oriented (Zhu, 2011; Wen et al., 2013; Zhang, 2018). Meanwhile, more consideration should be given to motivating students' initiative for learning rather than on teacher's lecturing (Ding, 2016). Furthermore, international students could be given intercultural trainings before and after they come to China in order to obtain necessary skills and positive motivations to live in an intercultural environment.

## Conclusions

To conclude, this study has investigated motivations of 671 international students dynamically by looking into two different phases (preliminary phase and follow-up phase) and offered explanations in their choice of China as study destination and their motivational adjustments during their academic journey in China. Analysis of data from surveys suggested that an integrative view is essential to comprehend international students' motivations to China. International students are diversified in their motivations, and motivation is not the sole pulling force (Wen and Hu, 2019). Economic and sociocultural motivations could also attract international students. In addition, prospective students are driven more by intrinsic self-motivated pushing force than the external pulling force in selecting China as their study destinations. Moreover, the longitudinal data analysis of reflective journals and interviews has shown that a dynamic perspective is necessary in understanding how international students sustain their academic journey by making relative motivational adjustments when they encounter difficulties and problems in China. The positive interaction between international students and the host society is more likely to produce long-lasting intrinsic driving forces that motivate students to obtain academic success and achieve sustainable personal development. Consequently, the government, universities and educational practitioners should create more favorable conditions to facilitate international students in order to better adjust to the Chinese academic environment (Ma and Wen, 2018; Gong et al., 2020) and stimulate their positive motivations.

While there are valuable and insightful contributions, this study is also limited in the following respects. Sample size is a major challenge that most empirical research in this field is facing. Thus, caution must be given when generalizing research findings to other international students in Chinese universities. Therefore, scholars are encouraged to carry out joint research in the future to collect large-scale samples with diverse nationalities, age groups or degree levels, and thus research findings could be more generalizable (Wen and Hu, 2019). Apart from that, most of the research participants are international students enrolled in degree programs of Chinese universities in Zhejiang province, while international students in non-degree programs and other areas of China are less emphasized. As a result, more attention could be given to those students in the future research (Wu et al., 2019). Despite the mentioned limitations, we believe the findings of the current study can provide alternative insights for understanding motivations and motivational changes among international students in Chinese universities, and suggest ways for students and relative parties in keeping academic journey sustainable. In addition, considering the importance of motivational factors in sustaining international students' academic journey, more attention should be paid to student sojourners' academic adjustment status in the future research.

## Data Availability Statement

The raw data supporting the conclusions of this article will be made available by the authors, without undue reservation.

## Ethics Statement

The studies involving human participants were reviewed and approved by the Expert Review Committee of Ningbo University. Written informed consent to participate in this study was provided by the participants' legal guardian/next of kin.

## Author Contributions

YM developed the research idea and drafted the manuscript. YM and RW contributed equally to the collection and analysis of the research data. YM and HJ revised the manuscript. All authors have read and agreed to the published version of the manuscript.

## Funding

This research project was supported by Scientific Research Fund of Zhejiang Provincial Education Department (Project No. Y202045265) and MOE (Ministry of Education in China) Project of Humanities and Social Sciences (Project No. 20YJC630047).

## Conflict of Interest

The authors declare that the research was conducted in the absence of any commercial or financial relationships that could be construed as a potential conflict ofinterest.

## Publisher's Note

All claims expressed in this article are solely those of the authors and do not necessarily represent those of their affiliated organizations, or those of the publisher, the editors and the reviewers. Any product that may be evaluated in this article, or claim that may be made by its manufacturer, is not guaranteed or endorsed by the publisher.
